# Paraspinal muscle degeneration and regenerative potential in a Murine model of Lumbar Disc Injury

**DOI:** 10.1016/j.xnsj.2021.100061

**Published:** 2021-04-20

**Authors:** Michael R. Davies, Gurbani Kaur, Xuhui Liu, Francisco Gomez Alvarado, Prashant Nuthalapati, Mengyao Liu, Agustin Diaz, Jeffrey C. Lotz, Jeannie F. Bailey, Brian T. Feeley

**Affiliations:** University of California, San Francisco, Department of Orthopaedic Surgery, 1700 Owens Street, San Francisco, CA 94158, USA

**Keywords:** Multifidus degeneration, Fibroadipogenic progenitors, Murine model, Gait analysis

## Abstract

**Background Context:**

The association between low back pain and lumbar disc degeneration is not fully characterized. One potentially overlooked factor of this process is the lumbar multifidus, which plays a role in segmental stabilization and locomotion. Previous reports have shown multifidus degeneration to be associated with disc degeneration. The goal of this study was to develop a mouse model of advanced lumbar disc degeneration to recapitulate the pathology of the human multifidus in patients with lumbar disc degeneration and low back pain.

**Methods:**

C57BL/6 mice underwent a left anterolateral approach to the lumbar spine and disc puncture with a micro scalpel at L5/6 and L6/S1. Mice underwent behavioral analysis and functional gait testing. A subset of mice underwent 14T T2-weighted MRI to assess disc degeneration and paraspinal muscle quality. At 6 and 15 weeks, mice were sacrificed, and the multifidus muscles were harvested and divided into proximal and distal segments relative to disc injury. Histological analysis was performed to assess muscle degeneration, fiber type, and macrophage density. Fibroadipogenic progenitors (FAPs) were isolated for gene expression analysis with qPCR.

**Results:**

MRI demonstrated decreased intervertebral disc signal and paraspinal muscle atrophy at 6 weeks, with progressive degeneration and atrophy at 15 weeks. Disc injury resulted in delayed functional recovery and impaired gait. Histology demonstrated progressive multifidus fibrofatty degeneration between 6 and 15 weeks. CD68+ macrophage density was increased at 6 weeks but not 15 weeks. FAPs exhibited increased fibrotic and adipogenic gene expression at 6 weeks compared to sham with less of a difference in gene expression by 15 weeks.

**Conclusions:**

We have developed a mouse model of disc injury-mediated paraspinal muscle degeneration that recapitulates features of degenerative muscle pathology observed in patients with lumbar disc degeneration, and highlights the role of FAPs in mediating fibrofatty muscle degeneration after disc injury.

## Introduction

The lifetime prevalence of low back pain (LBP) is 65-80%, and this is frequently encountered in the setting of lumbar disc degeneration [1]. Prior studies have demonstrated that a link exists between lumbar disc degeneration, disrupted locomotor function, and pain, however the mechanisms linking these processes are not fully characterized [2], [3], [4], [5].

The lumbar paraspinal muscles are likely an important, but less studied factor of this pathologic process, as they serve a crucial role in vertebral segmental stability as well as global function [6], [7], [8]. Previous large animal studies have demonstrated that disc disruption results in localized muscle atrophy and fatty infiltration at the level of injury, while nerve root injury results in multiple levels of pathologic muscle changes distal to the injury [9], [10].

We have previously reported that the lumbar multifidus muscle of patients undergoing lumbar discectomy surgery demonstrated evidence of atrophy and fatty degeneration compared to muscle taken from an injured part of the body [11], [12]. These pathologic changes in human muscle samples have been found to correlate with an increase in fibroadipogenic progenitor (FAP) stem cells in injured muscle, as well as increased fibrotic and adipogenic gene expression among this resident muscle stem cell population [12]. FAPs have also been shown to possess latent regenerative potential when exposed to certain pharmacologic substances such as beta-3 adrenergic agonists and histone deacetylase inhibitors [13], [14], however their regenerative potential has yet to be characterized in the lumbar multifidus.

In order to further investigate the mechanisms underlying lumbar multifidus muscle degeneration, a clinically relevant small animal model in which molecular and cellular pathways can be studied, perturbed, and assessed with biochemical and functional testing is essential. In this study, we describe the development of a mouse model of lumbar disc injury-mediated paraspinal muscle degeneration that recapitulates the muscle pathology observed in human patients and may provide important insight into the cellular and functional mechanisms linking disc degeneration, paraspinal muscle degeneration, and low back pain.

We hypothesized that a disc injury would result in characteristic degenerative changes to the disc and local paraspinal musculature, with an increase in FAPs followed by an increase in muscle degeneration. Establishing this relationship would not only provide novel insight into the origins of the fat and fibrosis observed in patients with lumbar disc degeneration and low back pain, it would further identify future therapeutic avenues for treating paraspinal muscle degeneration by leveraging the regenerative potential of FAPs.

## methods

### Study Design

Three-month-old male wild-type mice (C57B/L6J, Jackson Laboratory Inc.) were randomly divided into disc injury (DI) and sham control groups (n = 10/group). The surgical injury model was based upon the lumbar disc puncture model described by Shi et al. [2]. Mice were chosen as the model organism for this study given their wide applicability for future genetic studies to further investigate pathways potentially identified in paraspinal muscle degeneration. Mice were sacrificed at 6 weeks (n = 12) and 15 weeks (n = 8). All surgical procedures and post-procedure behavioral and functional testing was approved by the institutional Animal Studies Subcommittee (IACUC), protocol 20-003-01, animal assurance number A3476-01. Outcomes assessed included MRI, combined behavioral testing, gait analysis, muscle histology, flow cytometry, and gene expression analysis.

### Surgical Approach

Lumbar disc puncture surgeries were performed in C57B/L6J mice that routinely have 6 lumbar vertebrae. Mice underwent a left ventral retroperitoneal approach to expose the L5-6 and L6-S1 disc spaces as described by Shi et al. [2]. The iliac crest was used as a marker of the L5-6 disc space, which was accessed by longitudinally spreading through the psoas muscle in order to protect the great vessels. A micro scalpel (PFM Medical) was used to perform an annulotomy and a direct anterior disc puncture to a depth of 1mm that was repeated 3 times at L5-6 and L6-S1 to simulate a severe degenerative response. Surgery was performed under loupe magnification by an orthopaedic surgeon with 7 years of animal surgery experience.

### Magnetic Resonance Imaging (MRI)

At each timepoint, a subset of mice was selected for post-mortem 14 Tesla (T) T2-weighted MRI axial and sagittal sequences to evaluate the L5-6 and L6-S1 disc spaces and surrounding paraspinal musculature (6 weeks: n = 4, 15 weeks: n = 2). Disc spaces were evaluated for loss of signal intensity on the T2-weighted sagittal images, and adjacent vertebral endplates were observed for evidence of bony changes. The paraspinal musculature was assessed on axial views for evidence of muscle atrophy or abnormal signal.

### Combined Behavioral Score

At 2 days (n = 4/group), 2 weeks (n = 4/group), 4 weeks (n = 4/group), 6 weeks (n = 4/group), 12 weeks (n = 4/group), and 15 weeks (n = 3/group) postoperatively, mice were assessed using a modification of the combined behavioral score originally described by Gale et al [15]. This behavioral score, originally designed to assess graded spinal cord injury in rats, combines observations of spontaneous and evoked motor patterns along with simple and complex reflexes to assess functional deficits of the lower extremities [15]. The score was adapted for this study using the following parameters, scored separately for each hindlimb: gross motor function, toe spread, extension withdrawal, and placing. Total scores ranged from “0” indicating intact motor function, to “60” indicating severe dysfunction.

### Gait Analysis

At 6 weeks (n = 4/group), 8 weeks (n = 4/group), 10 weeks (n = 4/group), and 12 weeks (n = 4/group) postoperatively, mice underwent DigiGait analysis (Mouse Specifics, Framingham, MA) to quantify changes in the gait patterns of the right and left hindlimbs as previously described [16], [17].

The parameter of max dA/dT, referred to here as the “loading rate,” and defined as the rate of change of the pawprint area that contacts the treadmill surface as the paw touches down, was analyzed as a primary marker of impaired gait. Decreased loading rate is indicative of decreased forced development during braking, and has been associated with poor muscle health and peripheral nerve dysfunction [18], [19].

### Muscle Harvesting and Histology

At 6 and 15 weeks, mice were sacrificed and bilateral lumbar multifidus muscles were harvested. Muscles were then divided into segments proximal to L5 and distal to S1 to isolate segments of multifidus above and below the injured disc levels. These segments were then further divided into samples to be used for histology and samples used for flow cytometry and subsequent gene expression analysis.

Samples used for histological analysis were then mounted in 5% tragacanth gum, flash frozen in liquid nitrogen-cooled isopentane, and sectioned to a thickness of 10µm. For immunofluorescence staining of CD68 (1:100, ThermoFisher), PDGFRA (1:50, ThermoFisher) and laminin (1:200, Sigma-Aldrich), slides were fixed in 4% paraformaldehyde, washed in 0.1% PBS-Triton X-100 solution (PBST), blocked in 5% BSA in 0.1% PBST for 1 hour, and incubated with primary antibodies at 4°C overnight. Slides were then rinsed and incubated in secondary antibody (1:300, Jackson ImmunoResearch Laboratories, Inc.) for 30 min at room temperature, stained with 4’,6-diamidino-2-phenylindole (DAPI) and mounted with Fluoromount G. For muscle fiber type analysis, samples were incubated in 0.3% PBST for 30 min, blocked with 5% BSA in 0.3% PBST, and incubated for primary antibodies for BA-D5 (Type I fibers, 1:100, Developmental Studies Hybridoma Bank), SC71 (Type IIA fibers, 1:100, Developmental Studies Hybridoma Bank), BF-F3 (Type IIB fibers, 1:100, Developmental Studies Hybridoma Bank), and laminin (1:200, Sigma-Aldrich) overnight. Slides were then rinsed and incubated in secondary antibody (1:300, Jackson ImmunoResearch Laboratories, Inc.) prior to being mounted with Fluoromount G. Fibrosis was identified using Masson Trichrome staining (American Mastertech), and intramuscular lipids were identified using Oil-red-O staining.

The fibrosis and fat indices were quantified using ImageJ (NIH) by dividing the amount of collagen or lipids identified on Trichrome or Oil-red-O, respectively, by the total tissue section area (n = 3 to 4 mice/group/timepoint), as previously described [13], [14]. Muscle fiber cross sectional area (CSA) was quantified using ImageJ (n = 3/group).

### Flow Cytometry for FAPs and qPCR

At 6 and 15 weeks, muscle segments from bilateral proximal and distal muscle samples relative to disc injury were pooled (6 weeks: n = 4; 15 weeks n = 3) for flow cytometry isolation of Sca1^+^/PDGFRα^+^/CD31^−^/CD45^−^/integrin α7^−^  FAPs as previously described [14].

FAPs were sorted into Triazol reagent (ThermoFisher) for total RNA isolation extraction. Quantitative reverse-transcription-polymerase chain reaction (qPCR) was performed using Fast SYBER Green Mastermix (Applied Biosystems) on a Viia7 Real Time Detection System (Applied Biosystems) to quantify expression of adipogenic and fibrotic differentiation markers (S1). 5ng of cDNA were used in each reaction. Expression levels of each gene were normalized to the housekeeping gene 18S.

Fold changes were calculated between the right distal muscle samples of the DI mice relative to sham controls using the double delta cycle threshold method. The right distal muscle sample was chosen for gene expression analysis to attempt to control for iatrogenic effects of the surgical approach on the left-sided paraspinal muscles.

### Statistical Analysis

Two-way, unpaired Student's t-tests were used for statistical analysis between groups. Prism 8 software (version 8.4.3, GraphPad Software, Inc.) was used for statistical testing and figure creation. Statistical significance was defined by *p* < 0.05. Data are presented as mean ± standard deviation of measurement.

## Results

### Summary of surgical outcomes

Twenty mice underwent DI (n = 10) or sham surgery (n = 10) without complications. One additional mouse allocated to sham surgery group died perioperatively due to bleeding related to the anterior surgical approach. All mice were monitored closely for the first 5 days after surgery for the development of cauda equina syndrome, with regular checks to ensure that they did not have signs of bladder distention or difficulty voiding. None of the mice were found to have bowel or bladder dysfunction following surgery. Immediately following surgery, all mice were noted to have a left hindlimb drop related to the left-sided approach. Recovery of hindlimb function varied between DI and sham mice as described below. All 20 mice survived to their intended timepoints.

### MRI

**Fig. 1:**
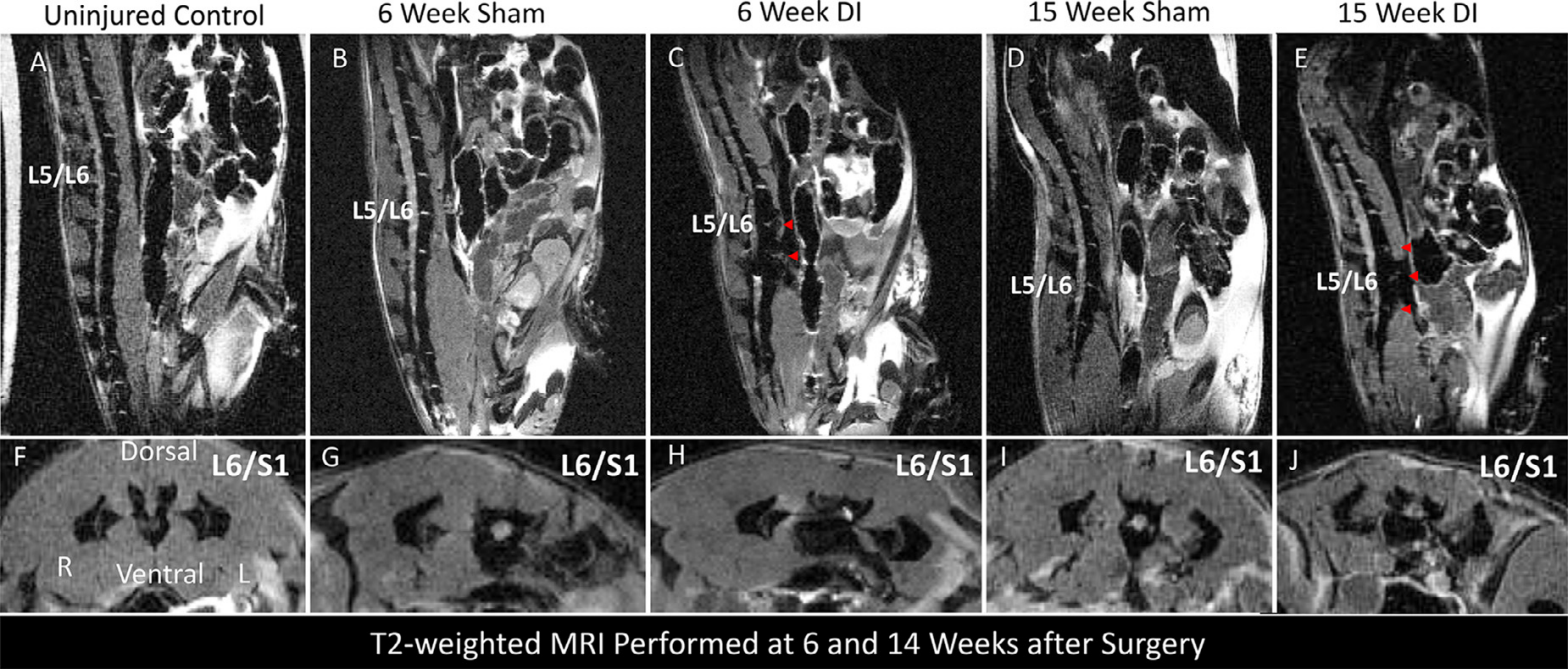
Disc injury results in progressive degenerative changes in the lumbar spine at 6 and 15 weeks. Representative T2-weighted sagittal (A-E) and axial (F-J) MRI images at 6 and 15 weeks obtained post-mortem using 14T MRI. Red arrows on sagittal images indicate severe disc degeneration characterized by loss of bright signal seen at uninjured levels.

Disc injury was found to lead to progressive degenerative changes seen on 14T MRI (n = 2/timepoint/group). 6 weeks after disc injury, there was loss of signal intensity at L5-6 and L6-S1 on MRI characteristic of disc degeneration [Fig. 1]. At L6-S1, there was additionally evidence of severe canal stenosis suggestive of progressive degenerative changes. These changes were not observed with sham surgery.

Compared to the uninjured control that did not undergo either sham surgery or DI, sham surgery resulted in unilateral left-sided paraspinal muscle atrophy appreciated on axial views, while DI resulted in bilateral paraspinal muscle atrophy compared to the uninjured control. By 15 weeks, there was evidence of advanced disc degeneration characterized by loss of signal at the L4-5, L5-6, and L6-S1 disc spaces seen on the T2-weighted mid sagittal image [Fig. 1].

On axial images, there was evidence of progression of paraspinal muscle atrophy bilaterally in the DI mouse compared to sham. Conversely, compared to 6 weeks, there appeared to be a relative increase in left paraspinal muscle volume in the sham mouse by 15 weeks.

### Behavioral analysis

**Fig. 2:**
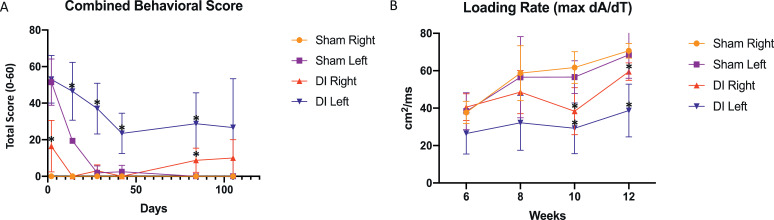
Disc injury results in delayed functional recovery compared to sham surgery. (A) Combined behavioral scores for hindlimbs of DI and sham mice, ranging from 0 indicating normal function, to 60 indicating maximal dysfunction as assessed by the scale. T-tests were performed between corresponding DI and Sham hindlimbs at each timepoint, with *p < 0.05 (n = 4 mice/group). (B) Loading rate, defined by max dA/dT measured by DigiGait software for hindlimbs of DI and Sham mice. T-tests were performed between corresponding DI and Sham hindlimbs at each timepoint, * denotes *p* < 0.05.

DI mice exhibited persistent functional impairment and abnormal gait compared to sham mice (n = 4/group/timepoint). Using the combined behavioral score described by Gale et al. [15], DI mice were found to exhibit impaired function at each timepoint assessed compared to sham mice [Fig. 2]. The left hindlimb of both groups demonstrated delayed recovery compared to the right side at each timepoint through week 6. By 12 weeks, DI mice had an average combined behavioral score of 28.8 ± 16.9 in the left hindlimb (*p* = 0.01) and 8.8 ± 6.6 (*p* = 0.04) in the right hindlimb, compared to scores of 0 in both the left and right hindlimbs of sham mice, indicating uniform recovery in the sham group by this time point.

### Gait analysis

**Fig. 3:**
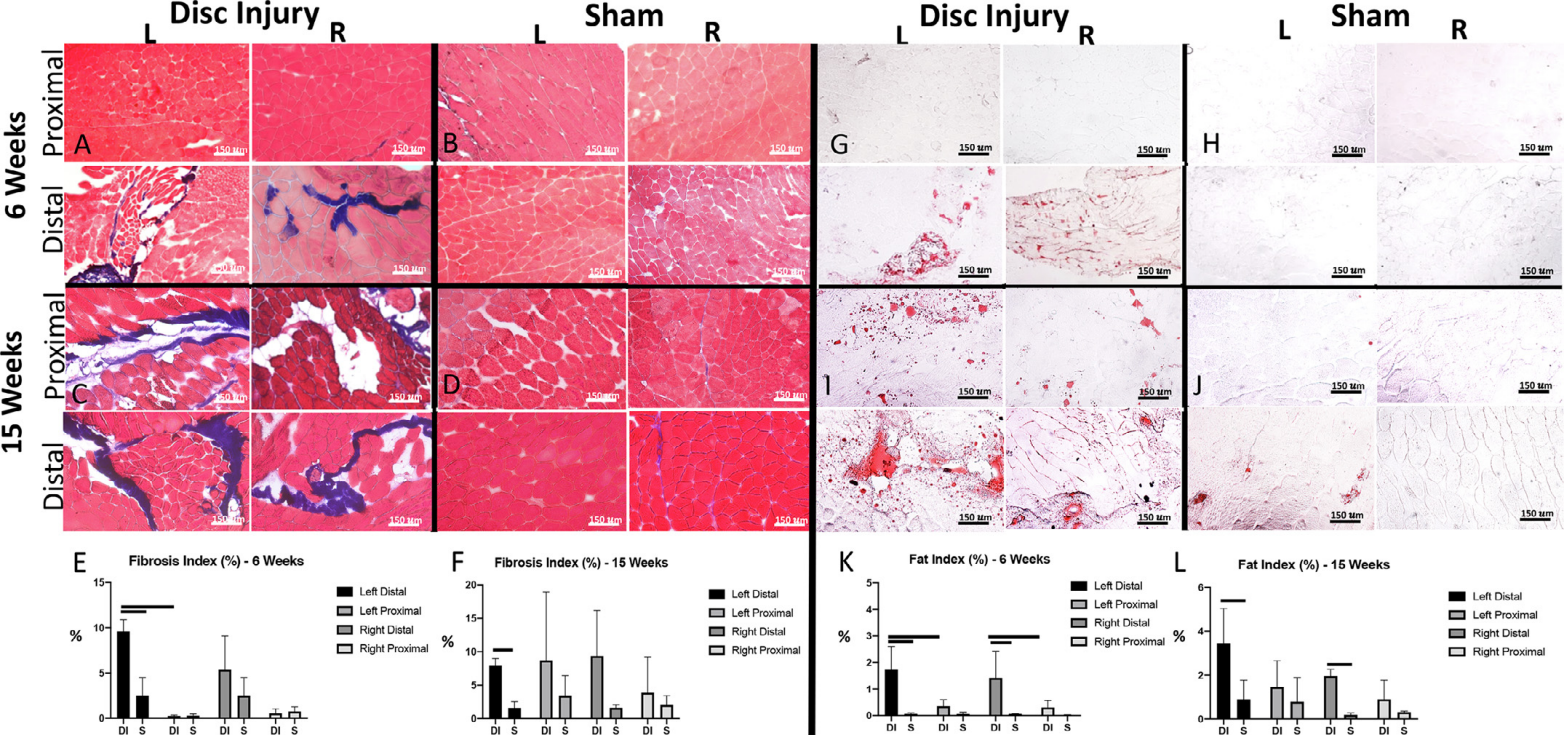
Disc injury leads to progressive fibrofatty degeneration of paraspinal muscle. (A-B) Representative Masson trichrome stains of proximal and distal muscle segments from DI and sham mice at 6 weeks and (C-D) 15 weeks. (E-F) Fibrosis Index calculated at 6 weeks and 15 weeks using ImageJ (n = 3–4 mice/group at 6 weeks, n = 3 mice/group at 15 weeks; 10x magnification; scale bar = 150μm). (G-H) Representative Oil red-O stains of DI and Sham mice at 6 weeks and (I-J) 15 weeks. Fat Index calculated at 6 weeks and 15 weeks using ImageJ (n = 3–4 mice/group at 6 weeks, n = 3 mice/group at 15 weeks; 10x magnification; scale bar = 150 μm). T-tests were performed between corresponding DI and sham muscle segments as well as and proximal and distal segments of within group; bar indicates *p* < 0.05.

Gait analysis demonstrated abnormal gait in DI mice compared to sham (n = 4/timepoint/group). DI mice exhibited decreased loading rate of both the left and right hindlimbs compared to sham by 10 weeks after injury (left DI: 29.2 ± 13.5 cm^2^/s vs. left sham: 56.5 ± 8.8 cm^2^/s, *p* = 0.02; right DI: 38.4 ± 12.6 cm^2^/s vs. right sham: 61.7 ± 8.8 cm^2^/s; *p* = 0.02). This significant decrease in loading rate in DI mice relative to sham persisted by 12 weeks after injury (*p* = 0.02 and *p* = 0.01 for left and right sides, respectively). The left hindlimb of DI mice demonstrated the lowest mean loading rate (max dA/dT) across all timepoints (Fig. 3).

### Evaluation of gross muscle histology

Disc injury led to progressive fibrofatty degeneration of the lumbar multifidus (n = 3-4/group/timepoint). By 6 weeks after injury, DI mice had developed evidence of muscle fibrosis and fatty infiltration in the lumbar multifidus muscle just distal to the level of disc injury. Fibrosis was significantly worse in the left distal multifidus of DI mice compared to sham, with a fibrotic index of 9.6% ± 1.3% compared to 2.5% ± 2.0% (*p* = 0.007). The mean fibrosis index of the right distal multifidus muscle of DI mice was elevated compared to sham, but did not reach statistical significance (5.8% ± 3.7% compared to 0.65% ± 1.3%, *p* = 0.1). There was significantly worse fatty infiltration of the bilateral lower multifidus muscle in DI mice (left: 1.8% ± 0.9% compared to 0.06% ± 0.04%, *p* = 0.02; right: 1.4% ± 1.0% compared to 0.06% ± 0.02%, *p* = 0.04).

At 15 weeks, DI mice continued to exhibit increased fibrosis of the left distal multifidus compared to sham ( 7.9% ± 1.1% compared to 1.6% ± 0.9%, *p* = 0.009). There was an increase in fibrosis of the muscle segment proximal to L5 such that by 15 weeks, no significant difference was noted in fibrosis between muscle taken proximal and distal to the levels of disc injury. There remained a significant increase in fatty infiltration of bilateral distal multifidus muscles in DI mice compared to sham, with the left side demonstrating the highest mean fat index (left: 3.4% ± 1.7% compared to 0.62% ± 0.46%, *p* = 0.048; right: 2.0% ± 0.3% compared to 0.19% ± 0.15%, *p* = 0.0009). Similarly, there was evidence of early fatty infiltration of the proximal muscle segments bilaterally in DI mice, such that there was no significant difference in fat index between proximal and distal muscle segments in DI mice, though distal muscle continued to exhibit a greater mean fat index.

### Evaluation of muscle atrophy and fiber type changes at 6 weeks

**Fig. 4:**
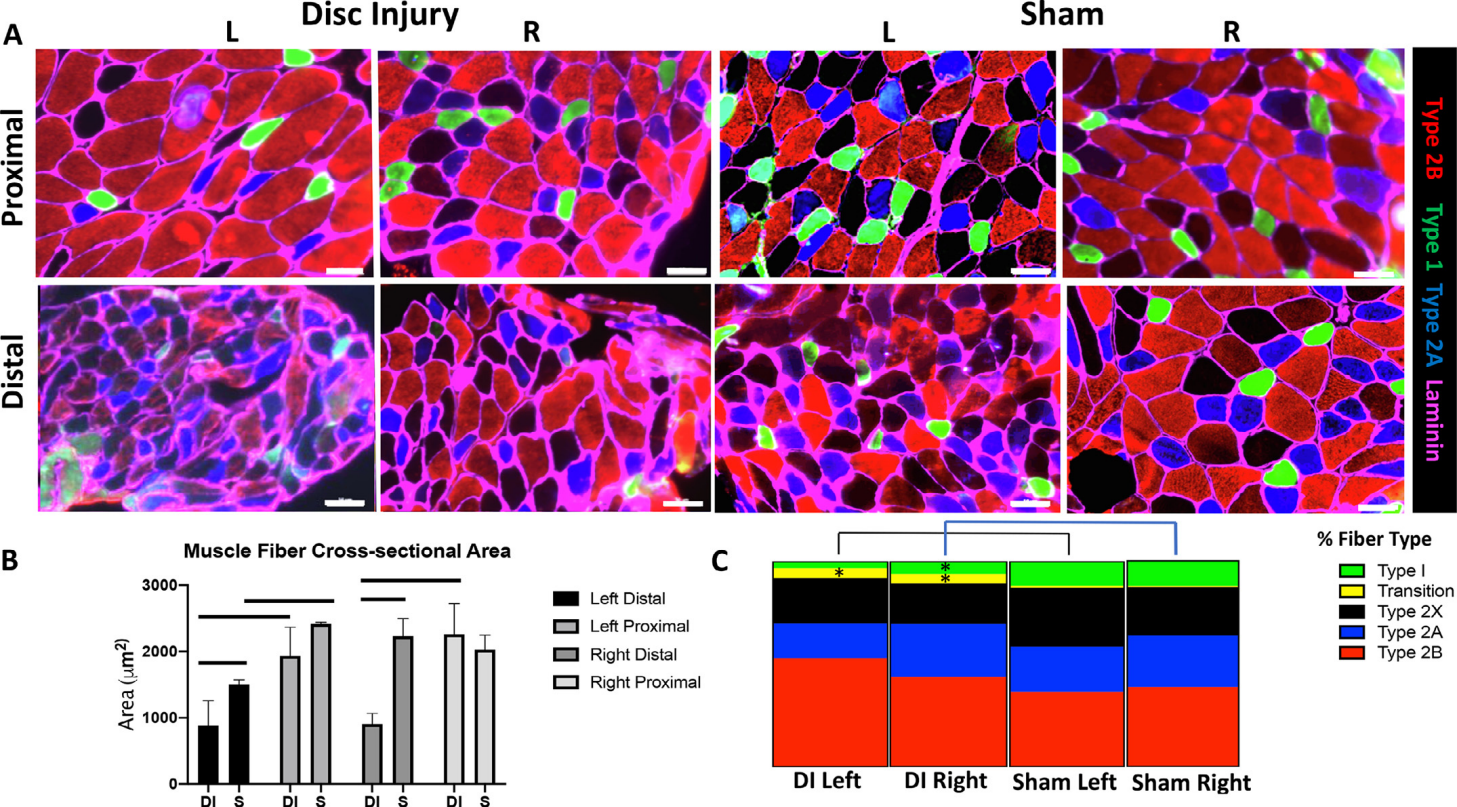
DI results in decreased fiber size and increased transition fibers at 6 weeks after injury. (A) Representative fiber type immunofluorescence at 6 weeks (20x magnification; scale bar = 50 μm). (B) Muscle fiber cross-sectional area calculated with ImageJ (n = 3 mice/group). (C) Quantification of fiber type indicated by immunofluorescence from distal muscle segments of DI and sham mice (n = 3 mice/group). T-tests were performed between corresponding sides of DI and sham muscles, *p < 0.05.

Multifidus fibers from DI mice displayed decreased cross-sectional area and changes in fiber type compared to sham mice at 6 weeks (n = 3/group). Given prior reports of muscle fiber type changes observed in the multifidus following a large animal disc puncture model, we elected to perform fiber size and type analysis at 6 weeks, corresponding with significant differences observed in the extent of fibrofatty degeneration between groups [20], [21]. There was a significant decrease in average muscle fiber CSA in the distal segment of bilateral muscles from DI mice (left: 885.3 μm^2^ ± 372.6 compared to 1505.9 μm^2^ ± 68.8 μm^2^, *p* = 0.048; right: 906.1 μm^2^ ±  160.8 μm^2^ compared to 2228.4 μm^2^ ± 268.9 μm^2^ , *p* = 0.002) [Fig. 4A, B]. There was also a significant decrease in the left distal multifidus muscle fiber CSA of sham mice compared to the ipsilateral proximal muscle (1505.9 μm^2^ ± 68.8 μm^2^ compared to 2412.6 μm^2^ ± 29.2 μm^2^, *p* < 0.0001).

Fiber type staining was performed to characterize the prevalence of type I, type IIa, type IIb and type IIx muscle fibers, as previous studies of paraspinal muscle degeneration have found that loss of type I muscle fibers may accompany lumbar disc degeneration and chronic low back pain [20], [21], [22], [23]. We found that the left distal multifidus muscle of DI mice demonstrated the greatest number of transition fibers at 9.5% ± 2.1%, defined by co-expression of BA-D5 (type I marker) and SC-71 (type IIa marker), significantly higher than the number observed in the left distal muscle of the sham group (*p* = 0.002) [Fig. 4A, C]. The right distal muscle of DI mice demonstrated a significantly higher number of transition fibers and significantly lower number of type I fibers compared to the right distal muscle of sham mice (*p* = 0.048 and 0.03, respectively).

### Macrophage density

**Fig. 5:**
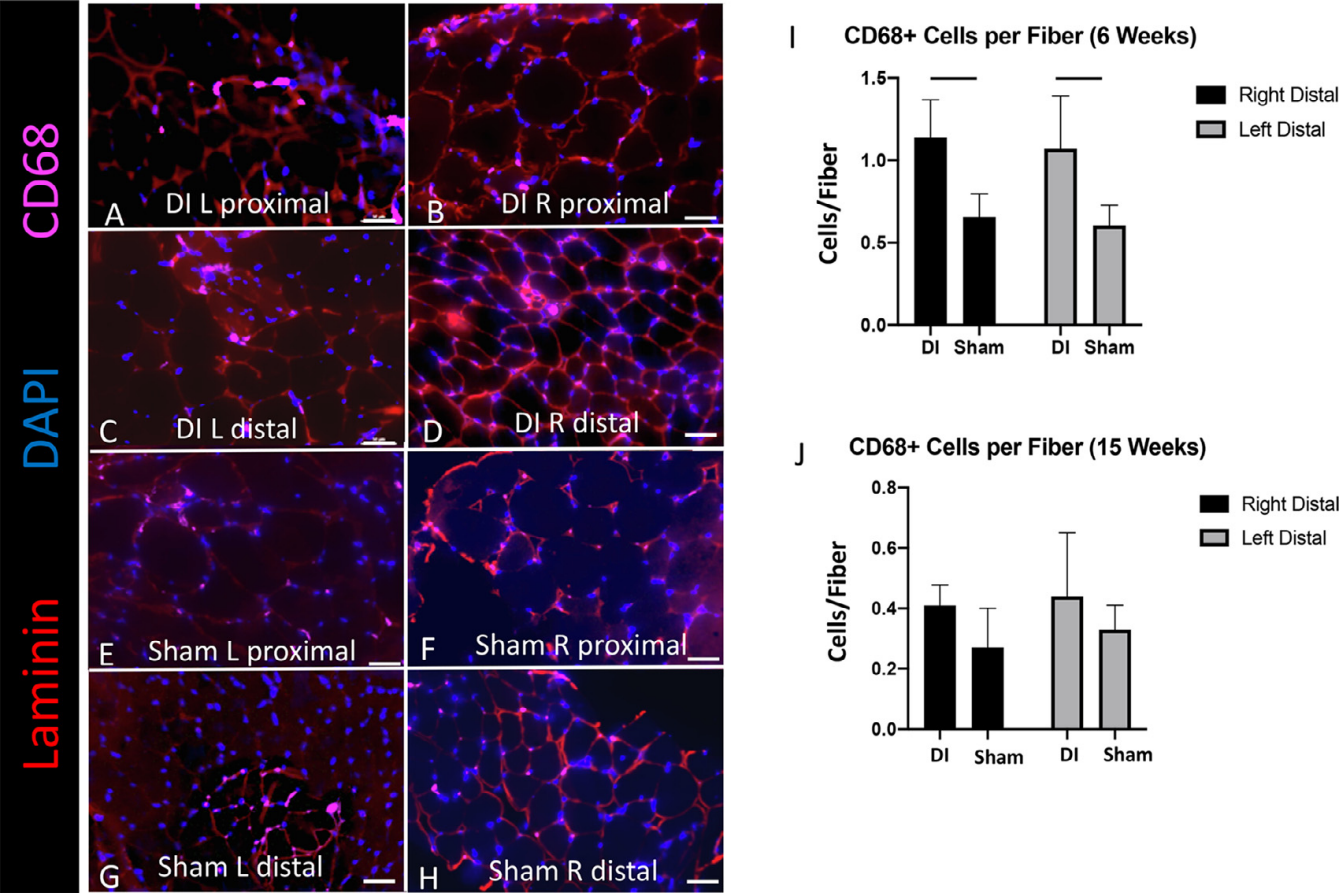
DI is associated with increased macrophage density at 6 weeks. (A-H) Representative CD68+ macrophage immunofluorescence at 6 weeks (20x magnification; scale bar = 50 μm). (I-J) CD68+ macrophage density per muscle fiber (n = 4 mice/group). T-tests were performed between corresponding sides of distal DI and sham muscle segments, *p < 0.05.

Macrophage density was increased in the multifidus of DI mice at 6 weeks after injury (n = 4/timepoint/group). As prior studies have implicated inflammatory cytokines such as TNF-α in the development of paraspinal muscle pathology in the setting of disc degeneration [20,[24], [25]], we sought to characterize the macrophage density among muscle fibers. At 6 weeks, we observed a significantly higher number of CD68+ macrophages per muscle fiber in the distal DI muscle compared to sham (left: 1.1 ± 0.3 compared to 0.6 ± 0.1, *p* = 0.03; right: 1.1 ± 0.2 compared to 0.7 ± 0.1, *p* = 0.01) [Fig. 5A-I]. By 15 weeks, the number of macrophages per fiber had decreased among all groups, and there was no significant difference in the macrophages per fiber between distal DI muscle compared to sham.

### Fibroadipogenic progenitor number and gene expression profile of sorted cells

**Fig. 6:**
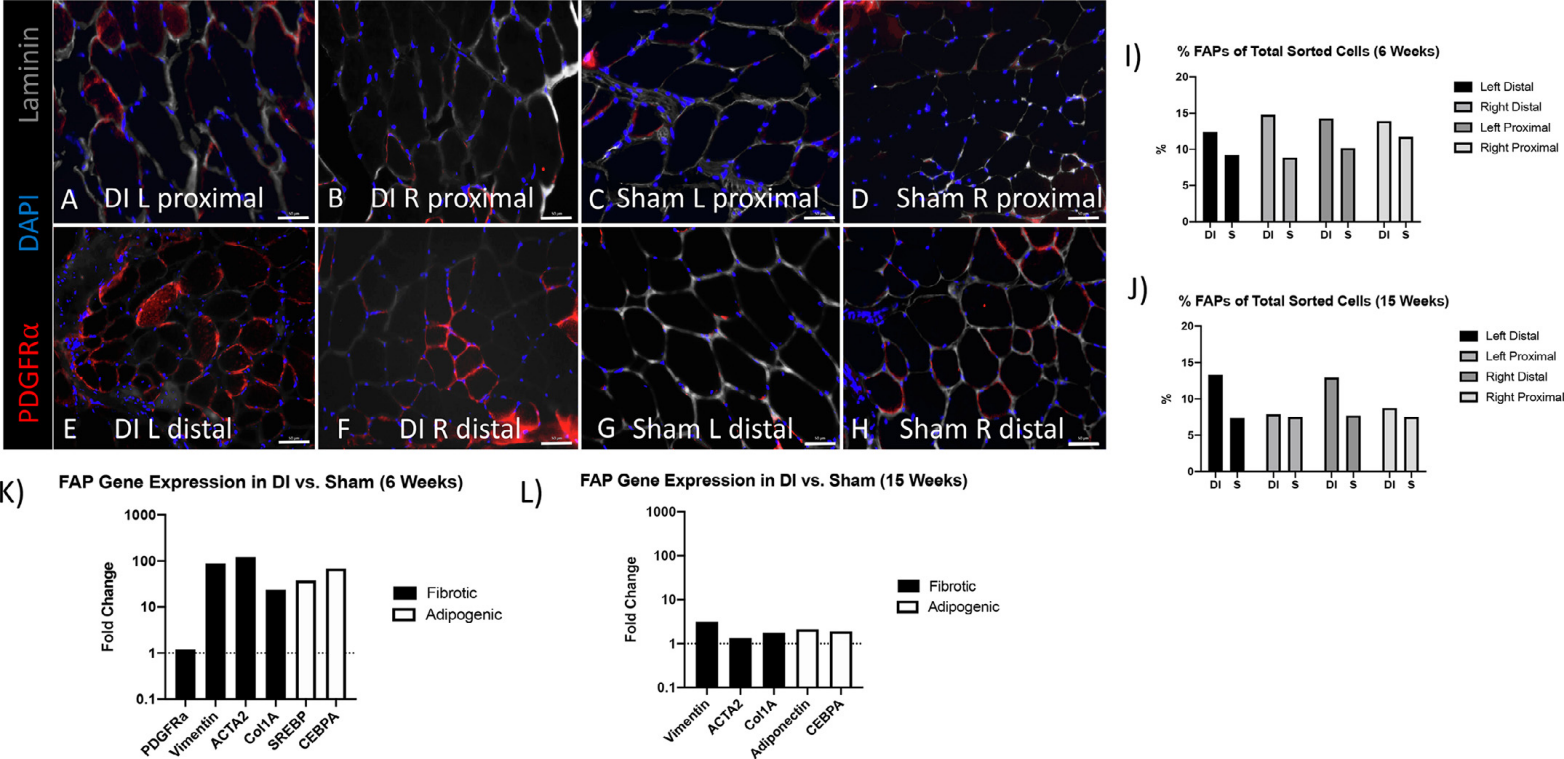
DI is associated with FAP fibrotic and adipogenic gene expression. (A-H) Representative PDGFRα FAP immunofluorescence at 15 weeks (20x magnification; scale bar = 50 μm). (I-J) FAP percentage of total cells sorted from pooled muscle samples taken at 6 weeks (n = 4/group) and 15 weeks (n = 3/group). (K-L) qPCR of fibrotic (Vimentin, ACTA2, Col1A) and adipogenic (SREBP, Adiponectin, CEBPA) differentiation markers at 6 weeks (K) and 15 weeks (L).

Given the development of marked atrophy, fibrosis, and fatty infiltration of the lumbar multifidus following disc injury, we quantified the number of FAPs and their gene expression patterns within the multifidus muscle. Given the necessity of dividing muscle segments into those that would be used for histological analysis as well as those used for flow cytometry, we proceeded to pool muscle samples from mice at 6 (n = 4) and 15 (n = 3) weeks in order to ensure a sufficient cell number for subsequent RNA extraction and qRT-PCR. Fig. 6A-H demonstrates representative histology of FAPs within muscle segments at 15 weeks after injury. FAPs remained elevated in the bilateral distal muscle segments of DI mice at 15 weeks [Fig. 6I, J].

At 6 weeks, we observed a 20-to-120 fold increase in markers of fibrotic (Vimentin: 87.5, ACTA2: 123.2, Col1A 23.7) and adipogenic (SREBP 37.7, CEBPA 68.2) differentiation in FAPs from distal DI muscle relative to distal sham muscle [Fig. 6K]. PDGFRA, a surface marker of FAPs, remained stable in expression between groups with a fold change of 1.2. By 15 weeks, differences in fibrotic and adipogenic gene expression had greatly decreased between FAPs from DI and sham groups [Fig. 6L].

## Discussion

In this study, we developed a mouse model of disc injury-mediated paraspinal muscle degeneration that recapitulates much of the muscle pathology frequently seen in patients with advanced lumbar disc degeneration and pathology, allowing for consistent detection of injury status and severity via diverse modalities including MRI, gait analysis, histology, and cell sorting with subsequent gene expression analysis. We have additionally demonstrated that FAPs likely have an important role in this degenerative process given their increase in adipogenic and fibrotic gene expression noted at 6 weeks, coinciding with worsening fibrofatty degeneration of muscle by 15 weeks. The implication of FAPs in the pathogenesis of lumbar multifidus degeneration is significant, because FAPs represent a large endogenous stem cell population within muscle that can be harnessed under the proper treatment conditions to improve muscle quality after injury [[13], [14],^17^,^26^].

An unintended but consistent consequence of performing a left-sided anterolateral approach to the lumbar spine was an iatrogenic left-sided nerve root injury, likely at the level of the lumbar plexus as it runs adjacent to the fibers of the psoas muscle, which were longitudinally spread in order to access the disc spaces of interest. Given the nature of the surgical approach, it is likely that this injury consisted of a neuropraxia from blunt dissection of psoas.  This process may have been exacerbated further by local inflammation from the surgical approach. We observed that mice in the sham surgery group displayed resolution of their left hindlimb dysfunction between 4–6 weeks as was evident with both behavioral testing as well as gait analysis, while mice that underwent disc puncture displayed persistent left hindlimb dysfunction for the remainder of the study that was greater than that of the right hindlimb. We propose that this finding likely resulted from multiple points of injury to the left-sided nerve roots in mice that underwent DI, although this warrants further investigation in future studies.

We observed a strong correlation between imaging findings, functional analysis, severity of findings on histology, and gene expression changes among FAPs. We found that in the sham group, left-sided unilateral paraspinal muscle atrophy observed both on MRI and histologic analysis at 6 weeks correlated well with lingering reduced function of the left hindlimb which subsequently resolved by 15 weeks, at which time neither MRI or histology demonstrated substantial pathologic changes. In the DI group, the bilateral MRI findings with evidence of concomitant lumbar disc degeneration at 6 and 15 weeks correlated well with bilateral hindlimb dysfunction observed on both behavioral and gait analysis, as well as increased severity of bilateral histological changes compared to sham at both timepoints.

Functionally, the results of gait analysis further cement the link between lumbar disc degeneration and chronic low back pain, and highlight the role of the paraspinal muscles as a potentially intervenable effect modifier. Gait analysis revealed a significantly decreased loading rate of both the left and right hindlimbs with disc injury. As the loading rate is a measurement of decreased force loading of the paw while braking, it may be indicative of pain, muscle weakness, and peripheral nerve dysfunction [18], [19]. Interestingly, the gradual improvement in loading rate with time among mice in both groups suggests that sham mice may have been continuing to show functional improvements in gait beyond 8 weeks out from surgery.

Mechanistically, inflammation and nerve root injury have both been previously studied as potential mediators of the muscle degeneration observed in the setting of lumbar disc degeneration. James et al. demonstrated that M1 pro-inflammatory macrophages were increased in the multifidus muscle of sheep that had undergone a disc lesion at 3 and 6 months after injury, correlating with an increase in expression of tumor necrosis factor (TNF) in adipose and connective tissue at 6 months, while total macrophage number remained stable over time [24].

Our findings support a role of macrophages in this process, but independently characterizing the contribution of nerve root injury to the observed muscle degeneration was beyond the scope of this study. We found that macrophage density among muscle fibers just below the disc lesions was significantly increased at 6 weeks compared to sham. By 15 weeks, no significant difference was observed between groups, and the number of macrophages per muscle fiber appeared reduced bilaterally in both groups. These findings support a temporal role of macrophage-mediated inflammation in the disc injury and subsequent muscle degeneration observed with this model. The fact that disc injury also resulted in the subtle appearance of fat and fibrosis above the level of the injured discs suggests that nerve root compression alone cannot account for paraspinal muscle degeneration in the setting of lumbar disc degeneration, and that inflammation may play an important role. Future studies are needed to better characterize the process of the proximal muscle degeneration observed. Additionally, given the role of macrophage polarization noted by James et. al [24], future experiments will assess the role of macrophage polarization in this injury model.

In other settings of muscle degeneration in which inflammation has been implicated, crosstalk between macrophages and FAPs has been highlighted as a key mechanism resulting in adipogenic and fibrotic differentiation of FAPs. Lemos et al. demonstrated that sustained TGF-β1 secretion from macrophages in chronic muscle injury states resulted in the survival and downstream adipogenic and fibrotic differentiation of FAPs [27]. Here, we observe changes in adipogenic and fibrotic gene expression of injured FAPs on the order of 20 to 120 fold at 6 weeks, correlating with a significant increase in macrophage density in DI muscle. While the percentage of sorted FAPs remains elevated among pooled samples at 15 weeks, we no longer observed large differences in adipogenic and fibrotic gene fold changes between groups at this time point. This correlated with a reduction in macrophage density in DI muscle that was no longer significantly elevated compared to sham. While this relationship warrants further investigation, we propose that macrophage-mediated inflammation may play an important role in activating FAPs to differentiate into fibroblasts and adipocytes in the setting of lumbar disc degeneration, resulting in the fibrofatty degeneration that is observed.

An interesting correlate with the presence of fibrofatty degeneration observed in this study was evidence of fiber type switching in disc injured muscle. At 6 weeks, we observed an increase in transition fibers in the bilateral distal DI muscle, as well as a loss of type I fibers, which was found to be statistically significant in the right-sided distal muscle. Previous studies have reported the presence of fiber type changes in the setting of both disc injury and low back pain. [20], [21], [22], [23]. As the mechanisms underlying fiber type switching in the setting of disc degeneration are not fully established, we aim to use this injury model to further characterize the processes underlying these changes in future studies.

This study has several limitations. While the muscle findings observed in mice do mimic those seen in cases of lumbar disc degeneration in patients, we recognize that the injury mechanism leading to these changes is acute in nature. However, given the advanced degenerative changes seen on MRI, the persistently antalgic gait observed functionally, and the advanced fibrofatty degeneration observed histologically, we propose that this model results in an accelerated process of muscle degeneration that may be used to study the same mechanisms that underlie paraspinal muscle degeneration in humans. Next, we acknowledge that for gene expression analysis, the need to use pooled samples in order to isolate a sufficient quantity of RNA for gene expression did not allow for the use of multiple biological replicates per time point. However, the magnitude of the fold changes that we observed among pooled samples at 6 weeks was strongly suggestive of a biologically important difference. Finally, we note that although we observe certain interesting correlations between imaging findings, functional analysis, muscle histology, and gene expression, we cannot pinpoint a single mechanism underlying the degenerative changes observed. We believe that this model will be useful for future experiments that rigorously define the mechanisms connecting inflammation, nerve root injury, FAP proliferation and differentiation, muscle fiber type changes, and ultimately, function. Understanding these relationships will be an important step to improving the outcomes of treatments focused on low back pain.

## Conclusions

We have developed a mouse model of lumbar disc injury that results in reproducible functional, histological, cellular, and molecular changes similar to those seen in chronic low back pain patients with lumbar disc pathology and degeneration. This model will prove useful in future studies defining the mechanisms underlying paraspinal muscle degeneration. Ultimately, preservation of the lumbar paraspinal musculature in the setting of lumbar disc injuries may serve as an important effect mediator of the severity of low back pain associated with lumbar disc pathology and degeneration, and this model will allow for efficient and reproducible evaluation of future therapeutic approaches.

## Funding Disclosure Statement

The funding for this study was provided by NIH grant R56AR063705. Reviewing authors’ individual financial disclosures, we have not identified any relevant conflicts of interest to this specific study.

## Declaration of Competing Interest

Michael R. Davies: None

Gurbani Kaur: None

Xuhui Liu: None

Francisco Gomez Alvarado: None

Prashant Nuthalapati: None

Mengyao Liu: None

Agustin Diaz: None

Jeffrey C. Lotz: None

Jeannie F. Bailey: None

Brian T. Feeley: Editor-Current reviews musculoskeletal medicine Associate editor, J Shoulder and Elbow surgery

## Appendix Supplementary materials

Image, application 1

Image, application 2
