# Supplementary material for: Paraspinal muscle degeneration and regenerative potential in a Murine model of Lumbar Disc Injury
Source: N Am Spine Soc J. 2021 Apr 20;6:100061. doi: 10.1016/j.xnsj.2021.100061 (PMC8820077; doi:10.1016/j.xnsj.2021.100061)
Supplement: Supplementary file 1 [file mmc1.docx]

**S1. qPCR Primer Information**

| Gene Name | Primer Sequence |
| --- | --- |
| PDGFRA | Forward: AGAGTTACACGTTTGAGCTGTC  Reverse: GTCCCTCCACGGTACTCCT |
| Vimentin | Forward: CGTCCACACGCACCTACAG  Reverse: GGGGGATGAGGAATAGAGGCT |
| ACTA2 | Forward: CCGACCGAATGCAGAAGGA  Reverse: ACAGAGTATTTGCGCTCCGAA |
| Col1A | Forward: CAGCCGCTTCACCTACAGC  Reverse: TTTTGTATTCAATCACTGTCTTGCC |
| SREBP | Forward: AGCCGTGGTGAGAAGCGCAC  Reverse: ACTGCTGCTGCCTCTGCTGC |
| CEBPA | Forward: GCGGGAACGCAACAACATC  Reverse: GTCACTGGTCAACTCCAGCAC |
| Adiponectin | Forward: GTTCCCAATGTACCCATTCGC  Reverse: TGTTGCAGTAGAACTTGCCAG |
